# High-dose opioid utilization and mortality among individuals initiating hemodialysis

**DOI:** 10.1186/s12882-021-02266-5

**Published:** 2021-02-23

**Authors:** Matthew Daubresse, G. Caleb Alexander, Deidra C. Crews, Dorry L. Segev, Krista Lentine, Mara A. McAdams-DeMarco

**Affiliations:** 1 Department of Epidemiology, Johns Hopkins Bloomberg School of Public Health, 615 N. Wolfe Street W6033, Baltimore, MD 21205 USA; 2 Center for Drug Safety and Effectiveness, Johns Hopkins University, Baltimore, MD USA; 3 Division of General Internal Medicine, Department of Medicine, Johns Hopkins Medicine, Baltimore, MD USA; 4 Division of Nephrology, Department of Medicine, Johns Hopkins University School of Medicine, Baltimore, MD USA; 5 Division of Surgery, Department of Medicine, Johns Hopkins University School of Medicine, Baltimore, MD USA; 6 Department of Internal Medicine, Division of Nephrology, Saint Louis University School of Medicine, Saint Louis, MO USA

**Keywords:** Opioid, Dialysis, Mortality

## Abstract

**Background:**

Individuals undergoing hemodialysis in the United States frequently report pain and receive three-fold more opioid prescriptions than the general population. While opioid use is appropriate for select patients, high-dose utilization may contribute to an increased risk of death due to possible accumulation of opioid metabolites.

**Methods:**

We studied high-dose opioid utilization (≥120 morphine milligram equivalents [MME] per day) among adults initiating hemodialysis in the United States between 2007 and 2014 using national registry data. We calculated the cumulative incidence (%) of high-dose utilization and depicted trends in the average percentage of days individuals were exposed to opioids. We used adjusted Cox proportional hazards models to identify which opioid doses were associated with mortality.

**Results:**

Among 327,344 adults undergoing hemodialysis, the cumulative incidence of high-dose utilization was 14.9% at 2 years after initiating hemodialysis. Among patients with ≥1 opioid prescription during follow-up, the average percentage of days exposed to high-dose utilization increased from 13.9% in 2007 to 26.1% in 2014. Compared to 0MME per day, doses < 60MME were not associated with an increased risk of mortality, but high-dose utilization was associated with a 1.63-fold (95% CI, 1.57, 1.69) increased risk of mortality. The risk of mortality associated with opioid dose was highest in the first year after hemodialysis initiation.

**Conclusions:**

The risk of mortality associated with opioid utilization among individuals on hemodialysis increases as doses exceed 60MME per day and is greatest during periods of high-dose utilization. Patients and clinicians should carefully weigh the risks and benefits of opioid doses exceeding 60MME per day.

**Supplementary Information:**

The online version contains supplementary material available at 10.1186/s12882-021-02266-5.

## Background

Individuals undergoing hemodialysis frequently report moderate to severe pain [1, 2], and receive three-fold more opioid prescriptions than the general United States population [3]. Since 2007 over half of individuals on hemodialysis received at least one opioid prescription [3]. In 2010, almost one-quarter of individuals on dialysis received opioids for 90 or more days [4]. Together, these data suggest high-dose opioid utilization among hemodialysis patients may be common.

Between 1999 and 2011, aggressive marketing of prescription opioids and other forces, converged to drive unprecedented increases in opioid prescribing, addiction and overdose death in the United States [5–7]. While numerous investigations have examined these matters in the general population, fewer [3, 4, 8] have characterized the impact of the opioid epidemic among individuals with kidney disease, including those on hemodialysis. This is important because individuals with end-stage kidney disease (ESKD) may be likely to be prescribed high doses of opioids and those undergoing hemodialysis, in particular, face a disproportionately high risk of opioid-related adverse events [9]. For example, hydrocodone and oxycodone, which are the two most commonly prescribed opioids among adults undergoing hemodialysis [3], are recommended to be used with caution among patients with renal impairment due to the possible accumulation of parent drug and/or metabolites. We sought to expand upon prior investigations examining the impact of opioid utilization among adults undergoing hemodialysis by accounting for large variations in opioid doses across people and time.

The goals of our study were to: 1) characterize the prevalence of high-dose opioid utilization among individuals undergoing hemodialysis and 2) quantify the relationship between opioid dose and risk of death. A more thorough understanding of what doses of opioids may increase the risk of mortality may assist clinicians, patients and policy-makers in improving pain management and reducing mortality among hemodialysis patients in the United States.

## Methods

### Study population and data

We used national registry data from the United States Renal Data System (USRDS) which is linked to Medicare data to conduct a retrospective cohort study of patients initiating in-center hemodialysis treatment between January 1, 2007 to December 31, 2014. We included maintenance hemodialysis patients aged 18 years or age or older, who had no prior kidney transplant, and were enrolled in Medicare Parts A, B, (MPAB) and D. Each person contributed observation time from when they met all inclusion criteria until they died or experienced a censoring event, as defined below. We included individuals who met all inclusion criteria after the initiation of hemodialysis therapy as late entries; therefore, individuals who were late entries did not contribute time at risk until they meet all the inclusion criteria.

The USRDS uses a variety of data sources to compile sociodemographic, diagnostic, enrollment and treatment information for ESKD patients covered by Medicare. One of these sources, is the CMS ESKD Medical Evidence Report form (CMS2728). This form is used at the onset of ESKD to register patients, establish Medicare eligibility, and capture information on patient demographics and comorbidities. We supplemented the comorbidity data from the CMS2728 with claims from Medicare Part A and B. For each of the conditions listed in Supplemental Table 1, we considered either a report on the CMS2728 or an International Classification of Diseases, Ninth Revision (ICD-9) code in the year prior to hemodialysis initiation as evidence of the comorbid condition.

We obtained data on each person’s prescription drug claims from the Prescription Drug Event Standard Analytics Files in the USRDS. These data included dates of Part D enrollment, prescription dispense date, National Drug Code, quantity dispensed, strength, days supply, and dual-eligibility status for Medicare and Medicaid. In Supplemental Table 2, we list each of the opioids included in our analysis. We calculated Morphine Milligram Equivalents (MME) per day for each person using conversion factors from the Centers for Disease Control and Prevention (CDC), quantity of pills dispensed, days supply, and strength per pill [10].

### Study population of adults initiating hemodialysis

We used the data captured in the CMS2728 form and Medicare claims to characterize the sociodemographic factors and comorbidities of our study population. We examined the distribution of age, sex, race, ethnicity, dual-eligibility for Medicare/Medicaid, employment, cause of ESKD, Body Mass Index (BMI), comorbid conditions, and geographic region across the overall study period and by calendar year.

### Cumulative incidence of high-dose utilization

We defined high-dose opioid utilization, as greater than or equal to 120MME per day, which is consistent with the cutoff used by the Medicare Part D Overutilization Monitoring System implemented in 2013 [11]. We calculated the cumulative incidence of receiving any opioid prescription, a dose greater than 60MME per day, and high-dose utilization (≥120 MME per day) at 6 months, 1 year, and 2 years. We obtained estimates of the cumulative incidence function using the proc. lifetest procedure in SAS which allows for nonparametric analysis of competing risk data [12]. Individuals in this analysis contributed time from hemodialysis initiation until the first day of a period of high-dose utilization or censoring, which we defined as the end of hemodialysis and/or MPAB or Part D coverage, switch to peritoneal or home dialysis, transplant or end of follow-up. We accounted for death as a competing risk for the outcome of high-dose utilization.

### Prevalence of high-dose opioid utilization within one year of hemodialysis initiation

To describe the prevalence of high-dose opioid utilization within 1 year of hemodialysis initiation, we calculated the percentage of patients with one or more days of high-dose opioid utilization across sociodemographic factors. We used a chi-squared test to detect statistically significant differences between observed and expected frequencies.

### Trend of high-dose opioid utilization

We depicted trends in the annual average percentage of days exposed to each opioid dose category (0, 1 to < 30, 30 to < 60, 60 to < 90, 90 to < 120, and ≥ 120MME per day) among individuals on hemodialysis with at least one opioid prescription. We calculated the annual percentage of days each person was exposed to each dose by dividing the total number of days each person was exposed to each dose category in a given year by the total number of days they were observed on hemodialysis in that year. We then plotted the average percentage of days exposed at each dose category among all individuals observed in that year. To assess trends in the percentage of days exposed, we conducted separate linear regression models for each dose, where the outcome variable was a person’s percentage of days exposed at that dose and the independent variable of interest was year. In these models, we adjusted for age, sex, race, and Hispanic ethnicity.

### Opioid dose and mortality

To quantify the risk of all-cause mortality associated with opioid dose, we used a Cox proportional hazards model with a time-varying exposure. Individuals contributed observation time from hemodialysis initiation until they experienced the outcome of interest, death, or censoring. In our model, opioid doses were categorized using six levels of exposure: 0, 1 to < 30, 30 to < 60, 60 to < 90, 90 to < 120, or ≥ 120MME per day. Our final model adjusted for patient characteristics selected using the stepwise-AIC approach and a priori confounders (age, sex, race, ethnicity, dual-eligibility for Medicare/Medicaid, employment status, region, prior diagnoses for diabetes, and drug use). For each person, a Charlson Comorbidity Index Score [13], was included in the final model to control for channeling bias [14] because we expected individuals with a higher number of comorbidities to be more likely to require analgesia. We expected individuals with longer durations of prior opioid utilization to have developed either a tolerance to opioid analgesics or a higher-risk of death due to their need for long-term opioid analgesia. Therefore, to account for each person’s cumulative duration of opioid utilization, we included a time-varying running total of the number days exposed to opioids. We used Stata version 14 for variable selection and SAS version 9.3 for all other analyses.

### Sensitivity analyses

We conducted sensitivity analyses to assess the robustness of our results under different assumptions. In our analysis of the association between opioid dose and mortality, we first assessed the proportionality of our results before and after 365 days on hemodialysis. We re-ran our model among all individuals undergoing hemodialysis while censoring individuals still at risk after 365 days of follow-up (*N* = 327,344). We then ran our model only among individuals still at risk after 365 days (*N* = 200,117). We lacked Medicare claims data 1 year prior to hemodialysis initiation for approximately 25% of individuals in our study. To verify the inclusion of individuals lacking prior claims did not bias our results, we re-ran our model restricting our analysis to only individuals who had at least one Medicare claim 1 year prior to hemodialysis initiation (*N* = 245,107).

## Results

### Study population of adults initiating hemodialysis

Among the 327,344 adults initiating hemodialysis, 46% were female, 48% were aged > 65 years, 64% were white, and 52% were eligible for both Medicare and Medicaid (Table 1). Overall, the characteristics of our study population remained constant between 2007 and 2014.

**Table 1:** Characteristics of adults initiating hemodialysis between 2007 and 2014, by year (*N* = 327,344)

	Overall, % (***N =*** 327,344)	2007–2008, % (***N*** = 81,608)	2009–2010, % (***N*** = 87,646)	2011–2012, % (***N*** = 83,191)	2013–2014, % (***N*** = 74,899)
Age (years)					
18–35	4.4	5.2	4.8	4.2	3.2
36–50	14.7	16.0	15.6	14.5	12.2
51–65	33.2	33.5	33.7	34.1	31.0
> 65	47.8	45.2	45.8	47.2	53.6
Female	46.2	47.4	46.5	45.8	45.1
Race					
Black	31.0	33.4	31.8	30.1	28.4
White	63.8	61.4	62.8	64.7	66.6
Native American/Asian	5.0	4.9	5.2	5.1	4.8
Other/Unknown	0.2	0.3	0.3	0.2	0.1
Non-Hispanic	85.0	85.2	84.6	84.5	85.8
Not dual-eligible for Medicare/Medicaid	52.3	51.0	51.4	53.4	53.5
Employment					
Unemployed	24.7	24.1	24.7	25.3	24.5
Full or Part-time	5.7	7.0	6.4	5.3	3.9
Retired	38.7	36.8	37.1	38.2	43.1
Retired Disabled	25.8	25.6	26.0	26.3	25.2
Other	5.2	6.6	5.8	4.9	3.4
Cause of ESKD					
Diabetes	49.7	49.6	49.7	49.4	50.0
Hypertension	30.1	29.0	29.8	30.4	31.5
Other	20.2	21.3	20.6	20.2	18.5
Body Mass Index (kg/m^2^)					
< 18.5	2.6	3.0	2.6	2.5	2.5
18.5–25.0	29.0	30.4	28.8	28.3	28.3
25.0–35.0	27.8	27.9	27.7	27.7	27.9
≥ 30.0	40.6	38.7	40.8	41.4	41.3
Diabetes	65.7	65.5	66.8	66.3	64.1
Cerebrovascular	12.3	13.2	13.0	12.2	10.6
Peripheral Vascular	14.4	15.7	15.1	14.3	12.5
Hypertension	90.7	90.6	91.5	91.1	89.3
Chronic Obstructive Pulmonary	12.6	12.2	13.2	12.9	12.0
Tobacco Use	11.2	11.5	11.7	12.5	8.8
Cancer	8.8	8.9	9.3	9.0	7.7
Drug Use	1.9	2.2	2.0	2.1	1.5
Inability to Ambulate	10.5	10.3	11.3	11.3	8.9
Institutionalized	15.6	16.4	17.0	16.1	12.6
U.S. Geographic Region					
New England	3.4	3.5	3.5	3.4	3.4
Mideast	16.4	16.3	16.1	16.6	16.5
Great Lakes	15.7	15.4	15.4	15.7	16.4
Plains	5.5	5.8	5.6	5.3	5.3
Southeast	30.4	31.0	30.5	30.0	29.9
Southwest	13.3	13.3	13.3	13.3	13.1
Rocky Mountain	1.6	1.5	1.6	1.6	1.5
Far West	13.8	13.2	14.0	14.1	13.9

### Cumulative incidence and prevalence of opioid utilization

The estimated cumulative incidence of high-dose opioid utilization among all individuals initiating hemodialysis was 8.0% at 6 months, 11.3% at 1 year, and 14.9% at 2 years (Table 2). Among individuals with any opioid use, the incidence of high-dose utilization was 10.9% at 6 months, 15.1% at 1 year, and 19.5% at 2 years.

**Table 2:** Cumulative incidence of opioid utilization among adults initiating hemodialysis between 2007 and 2014 (*N* = 327,344)

	6 months	1 year	2 years
All hemodialysis patients			
Any opioid prescription, %	56.0	66.7	75.2
≥ 60MME* per day, %	22.1	29.5	36.9
≥ 120MME* per day, %	8.0	11.3	14.9
Hemodialysis patients with any opioid use			
≥ 60MME* per day, %	30.0	39.4	48.1
≥ 120MME* per day, %	10.9	15.1	19.5

*MME per day = ((quantity*strength)*opioid conversion factor)/days supply

Among individuals with one or more opioid prescriptions during follow-up, 14.4% received high-dose opioids within 1 year of hemodialysis initiation (Table 3). High-dose utilization was most common among individuals who were White (15.6%; *p* < 0.0001), non-Hispanic (15.1% vs. 10.5%; *p <* 0.0001) and engaged in prior drug (24.5% vs. 14.2%; *p <* 0.0001) or tobacco use (20.3% vs. 13.5%; *p <* 0.0001).

**Table 3:** Percentage of adults with ≥1 day of high-dose opioid utilization (≥120MME per day) within one year of hemodialysis initiation, 2007–2014 (*N =* 327,344)

	All hemodialysis patients (***N*** = 327,344)		Hemodialysis patients with ≥ 1 opioid prescriptions (***N*** = 232,546)	
	Overall, %	***p***-value*	Overall, %	***p***-value*
Prevalence of high-dose utilization	10.2	–	14.4	–
Age (years)				
18–35	12.1	< 0.0001	16.0	< 0.0001
36–50	13.0		17.0	
51–65	11.5		15.8	
≥ 66	8.3		12.3	
Sex				
Female	9.2	< 0.0001	13.6	< 0.0001
Male	11.4		15.2	
Race				
Black	9.5	< 0.0001	13.0	< 0.0001
White	11.0		15.6	
Native American/Asian	4.6		7.3	
Other/Unknown	9.1		14.2	
Ethnicity				
Non-Hispanic	10.7	< 0.0001	15.1	< 0.0001
Hispanic	7.4		10.5	
Medicare/Medicaid Eligibility				
Non-Eligible	8.6	< 0.0001	13.0	< 0.0001
Dual-Eligible	12.0		15.7	
Employment				
Unemployed	10.4	< 0.0001	14.2	< 0.0001
Full or Part-time	6.1		9.8	
Retired	8.3		12.4	
Retired Disabled	14.0		18.3	
Other	9.3		13.2	
Cause of ESKD				
Diabetes	10.6	< 0.0001	14.5	< 0.0001
Hypertension	8.6		12.4	
Other	11.8		17.1	
Body Mass Index (kg/m^2^)				
< 18.5	10.9	< 0.0001	16.2	< 0.0001
18.5–25.0	9.3		13.7	
25.0–35.0	9.3		13.3	
≥ 30.0	11.5		15.4	
Diabetes				
No	9.4	< 0.0001	13.8	<.0001
Yes	10.6		14.6	
Cerebrovascular				
No	10.2	0.09	14.3	0.05
Yes	10.5		14.8	
Peripheral Vascular				
No	9.8	< 0.0001	13.8	< 0.0001
Yes	12.9		17.6	
Hypertension				
No	10.7	< 0.01	15.7	< 0.0001
Yes	10.2		14.2	
Chronic Obstructive Pulmonary				
No	9.7	< 0.0001	13.7	< 0.0001
Yes	13.7		18.7	
Tobacco Use				
No	9.5	< 0.0001	13.5	< 0.0001
Yes	16.0		20.3	
Cancer				
No	9.9	< 0.0001	14.0	< 0.0001
Yes	13.0		18.5	
Drug Use				
No	10.0	< 0.0001	14.2	< 0.0001
Yes	19.2		24.5	
Inability to Ambulate				
No	9.9	< 0.0001	13.9	< 0.0001
Yes	12.8		18.9	
Institutionalized				
No	9.8	< 0.0001	13.7	< 0.0001
Yes	12.2		18.5	
U.S. Geographic Region				
New England	10.5	< 0.0001	15.5	< 0.0001
Mideast	7.9		12.8	
Great Lakes	11.3		15.7	
Plains	12.6		16.8	
Southeast	11.0		14.8	
Southwest	10.3		13.9	
Rocky Mountain	13.4		18.3	
Far West	8.4		12.2	

*chi-squared test of association

### Trend of high-dose opioid utilization

**Fig. 1:**
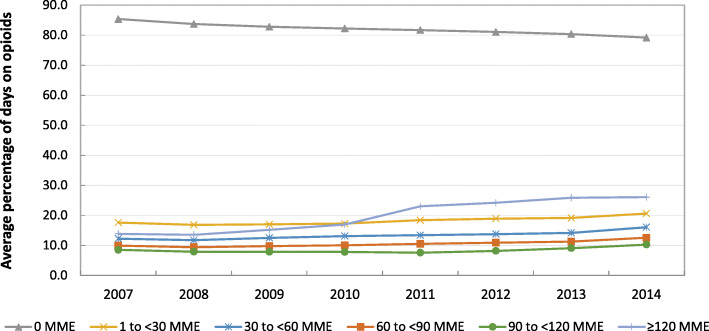
Average percentage of days on opioids by dose* of total days observed among adults on hemodialysis with at least one opioid prescription by year (*N* = 232,546). MME: Morphine milligram equivalents; SE: Standard error; CI: Confidence interval *Total days on opioid dose/total days observed; patients may contribute time in multiple years; **Linear regression parameter estimates for year, adjusted for age, sex, race, and Hispanic ethnicity

From 2007 through 2014, the average percentage of days exposed to each opioid dose category increased, whereas the percentage of days exposed to 0MME per day decreased (Fig. 1). The largest increase was in the average percentage of days exposed to high-dose utilization (2007: 13.9%; 2014: 26.1%) and doses 30 to < 60MME per day (2007: 12.2%; 2014: 16.0%). Between 2007 and 2014, the average annual increase in the percentage of days exposed to opioid doses ≥120MME per day after adjustment for age, sex, race, and Hispanic ethnicity was 2.1% (*p*-value: < 0.0001).

### Opioid dose and mortality among all individuals on hemodialysis

Individuals initiating hemodialysis who received doses from 1 to < 30MME per day (HR: 0.88; 95% CI: 0.85, 0.90) and 30 to <60MME per day (HR: 0.98; 95% CI: 0.95, 1.01) showed no increased risk in mortality compared to those receiving 0MME per day after adjusting for patient characteristics, comorbid conditions, and region (Table 4). When compared to no opioids, the risk of mortality increased once opioid dose exceeded 60MME per day. For example, doses between 60 and < 90MME per day were associated with a 1.30-fold (95% CI: 1.25, 1.34) increased risk of mortality. Compared to individuals receiving 0MME per day, individuals receiving ≥120MME per day had a 1.63-fold (95% CI, 1.57, 1.69) higher risk of mortality.

**Table 4:** Risk of mortality associated with opioid dose among adults initiating hemodialysis, 2007–2014^a^ (*N* = 327,344)

Dose (MME per day)	HR (95% CI)	***p***-value	***p***-value for trend
No opioids	REF	–	< 0.0001
1 to < 30	0.88 (0.85, 0.90)	< 0.0001	
30 to <60	0.98 (0.95, 1.01)	0.1162	
60 to < 90	1.30 (1.25, 1.34)	< 0.0001	
90 to < 120	1.29 (1.22, 1.36)	< 0.0001	
≥ 120	1.63 (1.57, 1.69)	< 0.0001	

^a^Cox proportional hazard model with time varying exposure for dose and adjustment for patient age, race, ethnicity, sex, employment status, cause of ESKD, body mass index, comorbid conditions (diabetes, cerebrovascular, arteriosclerotic heart disease, peripheral vascular, hypertension, chronic heart failure, chronic obstructive pulmonary, tobacco use, cancer, drug use, inability to ambulate, needs assistance, institutionalized, no prior comorbidities), Charlson comorbidity index, U.S. region, ESKD network, dual-eligible status for Medicare and Medicaid, incident year of hemodialysis, and cumulative days on opioids

### Opioid dose and mortality among individuals with one or more opioid prescriptions

In the subgroup of individuals with at least one opioid prescription, opioid doses exceeding 30MME were associated with an increased risk of mortality (Supplemental Table 3). Doses between 30 and < 60MME per day were associated with a 1.16-fold increased risk of mortality (95% CI: 1.13, 1.19) compared to 0MME per day. Compared to individuals receiving 0MME per day, the risk of mortality for individuals with high-dose utilization was 1.97-fold (95% CI: 1.90, 2.04) higher.

### Sensitivity analyses

For doses ≥60MME per day, the risk of mortality was highest in the first 365 days of hemodialysis. For example, in the first 365 days of hemodialysis, the risk of mortality for individuals with high-dose utilization was 1.78-fold (95% CI: 1.68, 1.89) higher compared to individuals receiving 0MME per day; however, after 365 days the risk of mortality associated with high-dose utilization was 1.46 (95% CI: 1.39, 1.52) (Supplemental Table 4). When we excluded individuals with no evidence of Medicare claims 1 year prior to hemodialysis initiation from the analysis, the association between opioid dosage and mortality remained consistent with the results in our main analysis (Supplemental Table 5).

## Discussion

In this retrospective study of 327,344 individuals initiating hemodialysis in the United States, we found 67% of adults undergoing hemodialysis received at least one opioid prescription within 1 year of hemodialysis initiation, and 11.3% of all patients experienced high-dose utilization. Among individuals with at least one opioid prescription, the average percentage of days with high-dose utilization increased from 2007 through 2014. Opioid doses ≥60MME per day were associated with an increased risk of mortality and the risk of mortality associated with opioid utilization is higher in the first year of hemodialysis.

Few prior studies have examined adverse outcomes associated with opioid use in the hemodialysis and peritoneal dialysis population [4, 8]. One study suggests that compared to no opioid use, both short-term and long-term opioid prescriptions were associated with a higher risk of death, dialysis discontinuation, and hospitalization [4]. Another retrospective cohort study found, among all individuals receiving hemodialysis in 2011, both doses lower than or equal to 60MME as well as doses greater than 60MME per day were associated with an increased risk of altered mental status, fall, and fracture when compared to no opioid use [8]. We add to previous literature suggesting short-term and long-term opioid prescriptions of varying doses were associated with mortality by also demonstrating the risk of dose-related mortality was highest within the first year after hemodialysis initiation. In addition, we found that compared to no opioid use, opioid doses less than 30MME were associated with a lower risk of mortality, which may be explained by increased engagement with healthcare providers or improved daily functioning as a result of adequate pain management.

Based on claims data from all individuals undergoing both hemodialysis and peritoneal dialysis in 2010, one prior retrospective study found that short-term (1–89 days) and chronic (≥90 days) opioid utilization of any opioid dose was associated with a 1.05 to 1.39-fold increased risk of all-cause mortality among dialysis patients [4]. However, this study was limited to prevalent dialysis patients and may have failed to account for patients who received opioids and died shortly after dialysis initiation. The authors found chronic utilization of doses greater than 50MME per day in a prevalent dialysis population were associated with a 1.39-fold increased risk of mortality. We found substantial increases in mortality risk up to doses of 120MME per day and higher among individuals initiating hemodialysis. Differences in the study period and methodological approach may explain, in part, why our results differ from this prior study.

This work highlights the importance of prescribing the minimum necessary dose of opioids for pain management among individuals undergoing hemodialysis, but should not deter providers from aiding patients in achieving adequate pain management. Between 2007 and 2014, almost three-quarters of individuals on hemodialysis in our study received at least one opioid prescription. Despite recent declines in opioid prescribing in the United States, we found the average percentage of days exposed to high-dose utilization increased among adults who received at least one opioid prescription during hemodialysis. Given opioid prescribing among individuals undergoing hemodialysis peaked between 2010 and 2012 [3], the increase in average percentage of days exposed to opioids observed from 2007 through 2014 is likely due to changes in socio-economic status and the growing percentage of individuals initiating hemodialysis who were white, retired, and over 65 years old. Prior work also suggests that between 2006 and 2010, the proportion of all individuals undergoing dialysis who had prescriptions for opioids ≥90 days increased from 20.0 to 23.4% [4]. These trends are alarming given oxycodone and hydrocodone, which are recommended to be used with caution or completely avoided in the hemodialysis population, are the two most commonly prescribed opioids among adults receiving hemodialysis. In 2014, HD patients were prescribed 333.9 MME of oxycodone per 100 person-days and 229.2 MME of hydrocodone per 100 person-days [3].

There are some important limitations to our study. First, we based our definition of utilization on prescription claims which does not fully capture quantity or duration of opioid consumption which is often prescribed pro re nata (PRN). This is a common limitation in claims-based research of opioids. Given the high prevalence of chronic pain among individuals undergoing HD and frequent encounters with dialysis staff, we assumed a high adherence to medicines to treat pain and thus calculated dose and duration using the quantity, strength, and days supply provided for each prescription claim. Second, we lacked data on the use of illicit opioids. Given only 2 % of the individuals in our study reported prior drug use and close to half were greater than 65 years of age, we suspect the lack of data on use of illicit opioids is unlikely to substantially affect our results. Third, we lacked data on opioid prescriptions prior to hemodialysis initiation which is likely associated with subsequent use. Fourth, in our analysis examining the association between high-risk opioid utilization and death, we were also concerned by channeling bias. We attempted to mitigate concerns related to these two limitations by including comorbid conditions and the Charlson Comorbidity index score in our models, restricting our analysis to patients with ≥1 opioid prescriptions, and adjusting for cumulative duration of opioid utilization. However, given the limitations of claims data and the inherent relationship between opioid dose and pathology, residual confounding as a result of channeling bias is possible despite these statistical adjustments. Finally, there are a number of MME per day cutoffs we could have used to define high-dose opioid prescribing [15, 16]. We attempted to address these limitations by using conversion factors from the CDC [10], which are commonly used for opioid research and selected a cutoff of 120MME per day which is equal to or higher than dosing thresholds commonly used in practice and research [17, 18].

Our analysis also had numerous strengths. First, we used a national registry with detailed information on individuals’ dialysis treatment, and sociodemographic characteristics. These data also provided us the strength, quantity, days supply and date of opioid prescriptions dispensed to our study population which allowed use to create a daily measure of opioid dose. Second, we accounted for the time-varying and reoccurring exposure of opioid utilization and quantified the increase in the risk of mortality between various opioid doses. Third, our results should be generalizable to the US population of individuals with ESKD because the majority enroll in Medicare and approximately 80% also enroll in Medicare Part D. Finally, by selecting hemodialysis initiation as our time origin, we were able to examine the risk of mortality associated with opioid dose during different periods of hemodialysis.

## Conclusions

In our study of adults initiating hemodialysis in the United States, we found 67% of individuals receive at least one opioid prescription and 11% receive at least 1 day of high-dose opioids within 1 year of hemodialysis initiation. Doses <60MME per day were not associated with an increased risk of mortality, but high-dose utilization was associated with a 1.63-fold increased the risk of mortality compared to 0MME per day. Our findings demonstrate the importance of minimizing opioid dose and improving the safety of pain management among individuals undergoing hemodialysis. Prescribers and individuals on hemodialysis should carefully weigh the benefits and risks of high-dose opioid treatment and even proceed cautiously with opioid doses as low as 60MME per day.

## Supplementary Information

**Additional file 1: Supplemental Table 1**. Methods for 1-year look back for claims data for comorbid conditions.

**Additional file 2: Supplemental Table 2**. Opioids included in analysis.

**Additional file 3: Supplemental Table 3**. Risk of mortality associated with opioid dose among adults on hemodialysis with ≥1 opioid prescriptions, 2007–2014* (*N* = 232,546).

**Additional file 4: Supplemental Table 4**. Risk of mortality associated with opioid utilization among adults initiating hemodialysis before and after 365 days, 2007–2014*.

**Additional file 5: Supplemental Table 5**. Risk of mortality associated with opioid dose among adults with ≥1 claims for comorbid conditions of interest one year prior to hemodialysis initiation, 2007–2014* (*N* = 245,107).

## Data Availability

The datasets generated and analyzed during the current study are available in the United States Renal Data System repository, https://www.usrds.org/for-researchers/standard-analysis-files/.

## Abbreviations

ESKD: End-stage kidney disease
USRDS: United States Renal Data System
MPAB: Medicare Parts A, B
CMS2728: CMS ESKD Medical Evidence Report form
ICD-9: International Classification of Diseases, Ninth Revision
MME: Morphine Milligram Equivalents
CDC: Centers for Disease Control and Prevention
BMI: Body Mass Index
CI: Confidence interval
