# Correction to: High-dose opioid utilization and mortality among individuals initiating hemodialysis

**DOI:** 10.1186/s12882-021-02332-y

**Published:** 2021-04-15

**Authors:** Matthew Daubresse, G. Caleb Alexander, Deidra C. Crews, Dorry L. Segev, Krista L. Lentine, Mara A. McAdams-DeMarco

**Affiliations:** 1 Department of Epidemiology, Johns Hopkins Bloomberg School of Public Health, 615 N. Wolfe Street W6033, Baltimore, MD 21205 USA; 2 Center for Drug Safety and Effectiveness, Johns Hopkins University, Baltimore, MD USA; 3 Division of General Internal Medicine, Department of Medicine, Johns Hopkins Medicine, Baltimore, MD USA; 4 Division of Nephrology, Department of Medicine, Johns Hopkins University School of Medicine, Baltimore, MD USA; 5 Division of Surgery, Department of Medicine, Johns Hopkins University School of Medicine, Baltimore, MD USA; 6 Division of Nephrology, Department of Internal Medicine, Saint Louis University School of Medicine, Saint Louis, MO USA

**Correction to: BMC Nephrology 22, 65 (2021)**

**https://doi.org/10.1186/s12882-021-02266-5**

Following publication of the original article [1], the authors identified an error in the author name of Krista L. Lentine.

The incorrect author name is: Krista Lentine

The correct author name is: Krista L. Lentine

The author group has been updated above. The original article has been corrected.
