# Supplementary material for: High-dose opioid utilization and mortality among individuals initiating hemodialysis
Source: BMC Nephrol. 2021 Feb 23;22:65. doi: 10.1186/s12882-021-02266-5 (PMC7901089; doi:10.1186/s12882-021-02266-5)
Supplement: Supplementary file 1 — Additional file 1: Supplemental Table 1. Methods for 1-year look back for claims data for comorbid conditions. [file 12882_2021_2266_MOESM1_ESM.docx]

**Supplemental Table 1. Methods for 1-year look back for claims data for comorbid conditions**

| **Comorbid Condition** | **ICD-9** |  |
| --- | --- | --- |
| Diabetes mellitus | 250.00 to 250.93 | |
| Cerebrovascular Disease | 430.XX to 438.XX, 430.0 to 438.0 | |
| Peripheral Vascular Disease | 443.0, 443.00:443.89 | |
| Hypertension | 401.9 | |
| Chronic Obstructive Pulmonary Disease | 491.20 to 491.22 | |
| Tobacco Use | 305.1 | |
| Cancer | 185.X, 193.X, 140.0 to 209.0 | |
| Drug Use | 303.00 to 303.03, 303.9, 303.90 to 303.93 | |
| Inability to Ambulate | 719.7 | |
| Institutionalized | Any claim in a nursing home or skilled nursing facility | |

For all 327,344 hemodialysis patients in the analytic cohort, we performed a 1-year look back in their Medicare Part A and B claims to supplement the comorbidity data from the 2728 form. Participants with the following ICD-9 code claims within 1 year prior to the start date of hemodialysis were considered to have the conditions listed above. We considered either a report on the 2728 form or an ICD-9 code in the year prior to hemodialysis initiation as the presence of the comorbid condition.
