# Supplementary material for: High-dose opioid utilization and mortality among individuals initiating hemodialysis
Source: BMC Nephrol. 2021 Feb 23;22:65. doi: 10.1186/s12882-021-02266-5 (PMC7901089; doi:10.1186/s12882-021-02266-5)
Supplement: Supplementary file 2 — Additional file 2: Supplemental Table 2. Opioids included in analysis. [file 12882_2021_2266_MOESM2_ESM.docx]

**Supplemental Table 2. Opioids included in analysis**

| Buprenorphine |
| --- |
| Butorphanol |
| Codeine |
| Dihydrocodeine |
| Fentanyl |
| Hydrocodone |
| Hydromorphone |
| Levomethadyl |
| Levorphanol |
| Meperidine |
| Methadone |
| Morphine |
| Opium |
| Oxycodone |
| Oxymorphone |
| Pentazocine |
| Propoxyphene |
| Tapentadol |
| Tramadol |
