# Supplementary material for: High-dose opioid utilization and mortality among individuals initiating hemodialysis
Source: BMC Nephrol. 2021 Feb 23;22:65. doi: 10.1186/s12882-021-02266-5 (PMC7901089; doi:10.1186/s12882-021-02266-5)
Supplement: Supplementary file 4 — Additional file 4: Supplemental Table 4. Risk of mortality associated with opioid utilization among adults initiating hemodialysis before and after 365 days, 2007–2014*. [file 12882_2021_2266_MOESM4_ESM.docx]

**Supplemental Table 4. Risk of mortality associated with opioid utilization among adults initiating hemodialysis before and after 365 days, 2007-2014***

|  | **<365 days**** | | **>365 days***** | |
| --- | --- | --- | --- | --- |
| **Dose (MME per day)** | **Hazard ratio (95% CI)** | **p-value** | **Hazard ratio (95% CI)** | **p-value** |
| No opioids  1 to <30  30 to <60  60 to <90  90 to <120  ≥120 | REF  0.92 (0.88, 0.95)  1.02 (0.98, 1.07)  1.40 (1.32, 1.48)  1.32 (1.22, 1.44)  1.78 (1.68, 1.89) | ---  <0.0001  0.3434  <0.0001  <0.0001  <0.0001 | REF  0.84 (0.81, 0.86)  0.92 (0.89, 0.95)  1.17 (1.12, 1.23)  1.21 (1.13, 1.30)  1.46 (1.39, 1.52) | ---  <0.0001  <0.0001  <0.0001  <0.0001  <0.0001 |

*Cox proportional hazard model with time varying exposure for dose and adjustment for patient age, race, ethnicity, sex, employment status, cause of ESKD, body mass index, comorbid conditions (diabetes, cerebrovascular, arteriosclerotic heart disease, peripheral vascular, hypertension, chronic heart failure, chronic obstructive pulmonary, tobacco use, cancer, drug use, inability to ambulate, needs assistance, institutionalized, no prior comorbidities), Charlson comorbidity index, U.S. region, ESKD network, dual-eligible status for Medicare and Medicaid, incident year of hemodialysis, and cumulative days on opioids.

******Data on hemodialysis patients still at risk after 365 days of follow-up were censored in this analysis. (N=327,344).

***Only hemodialysis patients still at risk after 365 days were included in this analysis (N=200,117)
