# Supplementary material for: High-dose opioid utilization and mortality among individuals initiating hemodialysis
Source: BMC Nephrol. 2021 Feb 23;22:65. doi: 10.1186/s12882-021-02266-5 (PMC7901089; doi:10.1186/s12882-021-02266-5)
Supplement: Supplementary file 5 — Additional file 5: Supplemental Table 5. Risk of mortality associated with opioid dose among adults with ≥1 claims for comorbid conditions of interest one year prior to hemodialysis initiation, 2007–2014* (N = 245,107). [file 12882_2021_2266_MOESM5_ESM.docx]

**Supplemental Table 5. Risk of mortality associated with opioid dose among adults with ≥1 claims for comorbid conditions of interest one year prior to hemodialysis initiation, 2007-2014* (N=245,107)**

| **Dose (MME per day)** | **Hazard ratio (95% CI)** | **p-value** |
| --- | --- | --- |
| Overall  No opioids  1 to <30  30 to <60  60 to <90  90 to <120  ≥120 | REF  0.87 (0.85, 0.90)  0.98 (0.95, 1.01)  1.32 (1.27, 1.37)  1.30 (1.23, 1.38)  1.62 (1.56, 1.69) | ---  <.0001  .1212  <.0001  <.0001  <.0001 |

*Cox proportional hazard model with time varying exposure for dose and adjustment for patient age, race, ethnicity, sex, employment status, cause of ESKD, body mass index, comorbid conditions (diabetes, cerebrovascular, arteriosclerotic heart disease, peripheral vascular, hypertension, chronic heart failure, chronic obstructive pulmonary, tobacco use, cancer, drug use, inability to ambulate, needs assistance, institutionalized, no prior comorbidities), Charlson comorbidity index, U.S. region, ESKD network, dual-eligible status for Medicare and Medicaid, incident year of hemodialysis, and cumulative days on opioids.
